# The role of Kras and canonical Wnt pathways for tumorigenesis of extrahepatic biliary system

**DOI:** 10.18632/oncotarget.28349

**Published:** 2023-01-26

**Authors:** Munemasa Nagao, Akihisa Fukuda, Hiroshi Seno

**Keywords:** ICPN, BilIN, biliary cancer, Kras, Wnt

Biliary tract cancer, including cholangiocarcinoma (CCA) and gall bladder carcinoma (GBC), accounts for approximately 3% of all adult malignancies [1]. WHO classification defined two representative precancerous lesions of CCA, biliary intraepithelial neoplasm (BilIN) and intraductal papillary neoplasm of the bile duct (IPNB) [2–4]. IPNB-like lesions in the gall bladder (GB) are defined as intracholecystic papillary-tubular neoplasm (ICPN) [5]. Cholangiocarcinoma is relatively rare, but annual mortality rates for CCA were >6 (per 100,000 inhabitants) in specific regions such as Japan, South Korea, China and Thailand [6]. Despite advances in diagnosis and therapy, 5-year survival rate of biliary cancer is only 5% to 15% [7, 8]. To develop novel therapeutic approaches, it is important to clarify the molecular mechanism underlying the development of biliary cancer and its precursor lesions.

Several whole-genome sequencing (WGS) studies have demonstrated that CCA is heterogeneous in terms of genetic mutations [9, 10]. WGS studies have revealed that the incidence of mutations in *KRAS* (16.5%) and WNT-related genes, including *APC* (7.1%), *RNF43* (4.7%), and *CTNNB1* (1.3%), are relatively frequent in biliary cancer [11]. Remarkably, the incidence of *KRAS* mutation is 31.5% in extrahepatic cholangiocarcinoma [12]. Mutations of *KRAS codon12* occurred in about 50% of early BilIN [13]. However, the role of the KRAS and WNT pathways in biliary tumorigenesis remained unclear.

In our recent paper, we investigated the role of the Kras and canonical Wnt pathways in the tumorigenesis of the extrahepatic biliary system including the extrahepatic biliary duct (EHBD) and GB by using a genetically engineered mouse (GEM) model [14]. At first, lineage tracing experiments revealed that *Hnf1b^CreER^* mouse is a suitable CreER mouse line with high efficiency for genetic manipulation in the extrahepatic biliary tract epithelium including the EHBD and GB. Next, we crossed *Kras^G12D^* mice and/or *Ctnnb1^lox(ex3)/+^* mice with *Hnf1b^CreER^* mice to generate *Hnf1b^CreER^(H), Hnf1b^CreER^*; *Kras^G12D^ (HK), Hnf1b^CreER^*; *Ctnnb1^lox(ex3)/+^ (Hβ)*, and *Hnf1b^CreER^*; *Kras^G12D^*; *Ctnnb1^lox(ex3)/+^ (HKβ)* mice. We found that concurrent activation of Kras and Wnt pathways in the adult mouse biliary epithelium induced biliary neoplasms that resembled human BilIN and gastric type ICPN in the EHBD and GB, respectively.

We next investigated whether BilIN and ICPN in *HKβ* mice were precancerous lesions. Because *HKβ* mice died due to massive intestinal adenomas, we established biliary spheroids derived from *H, HK, Hβ*, and *HKβ* mice and performed xenograft experiments. Notably, tumors of invasive biliary cancer developed in two out of 24 cases of *HKβ* biliary spheroids, whereas no xenograft tumors developed from *H, HK*, and *Hβ* biliary spheroids. These data indicated that ICPN and BilIN are precancerous lesions which progress to biliary cancer.

To investigate the molecular mechanism underlying the formation of ICPN and BilIN in *HKβ* mice, we next performed global gene expression analysis of biliary spheroids. In *HKβ* biliary spheroids, the expression of the Wnt/β-catenin signaling pathway, *c-Myc*, cancer stem cell markers (*Dclk1* and *Aldh1a1*), EMT pathway, and the Tgf-β signaling pathway was increased compared to *H, HK* and *Hβ* biliary spheroids.

Mechanistically, we found that c-Myc contributed to tumorigenesis in the context of activated Kras and Wnt signaling pathways. We showed that silencing *c-Myc* or pharmacological inhibition of c-Myc decreased the growth of *HKβ* biliary spheroids, suggesting that c-Myc was a critical mediator for biliary tumorigenesis in *HKβ* mice. Moreover, consistent with the mouse data, we observed upregulated Kras and Wnt signals and high c-MYC expression in human ICPN samples. On the other hand, we showed that concurrent activation of the Kras and Wnt pathways in the EHBD and GB resulted in upregulation of TGF-β pathway, which suppresses tumorigenesis and progression to biliary cancer. Notably, SMAD4 expression was decreased in human high grade BilINs [15]. Our data suggested that the TGF-β pathway is an important barrier for ICPN and BilIN to progress to biliary cancer and that TGF-β pathway may be a potential therapeutic target. Consistent with the mouse data, human gastric type ICPNs also displayed activated TGFβ pathways. In addition, TCGA database analysis of human biliary cancer indicated a correlative relationship between the expression of AXIN2 and MUC6, DCLK1, and the TGFβ pathway. These data further strengthen the human relevance of our mouse model.

**Figure 1:**
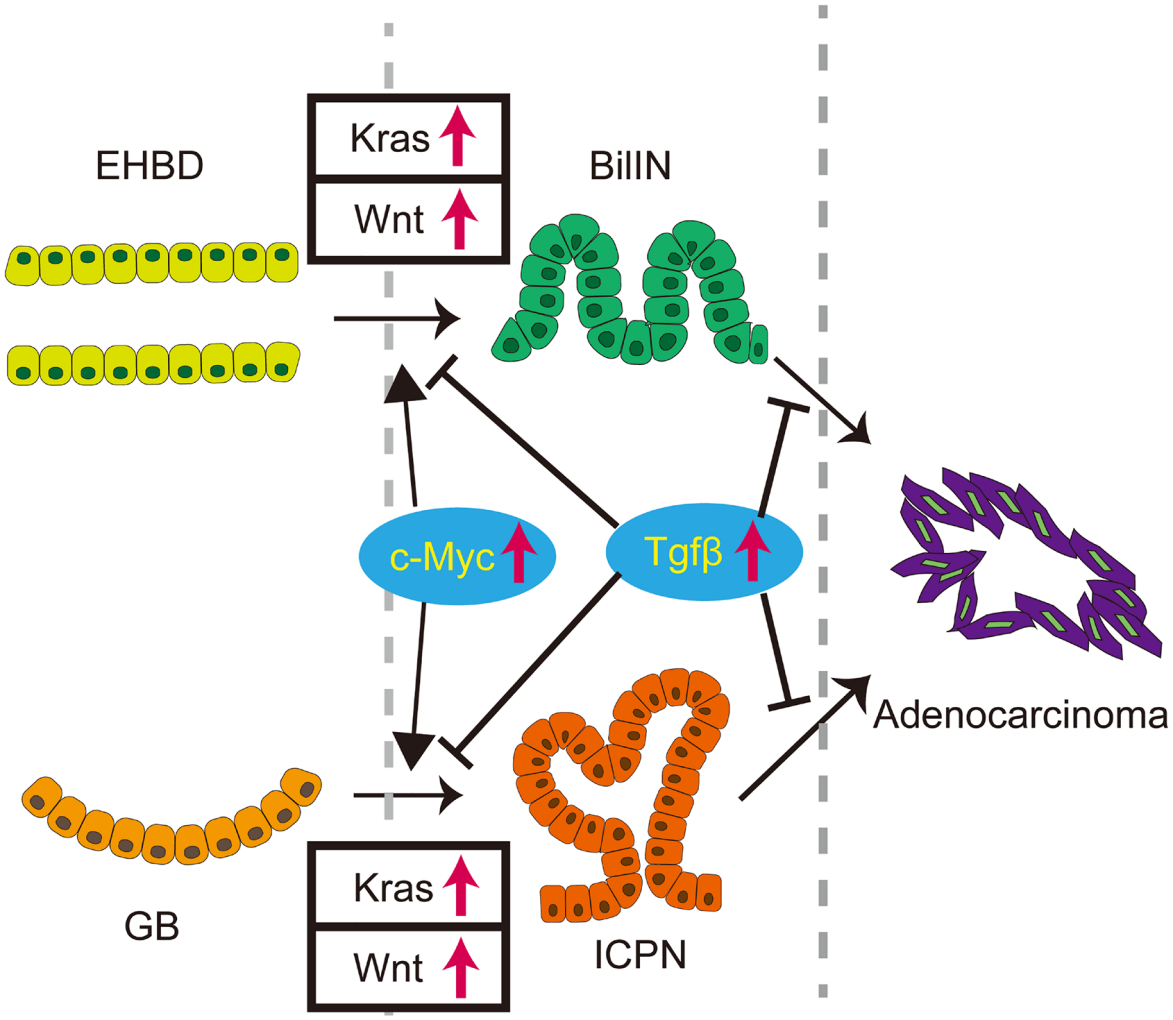
The role of Kras and canonical Wnt pathways for tumorigenesis of extrahepatic biliary system. Concurrent activation of Kras and Wnt pathways in the adult mouse biliary epithelium induces biliary neoplasms that resemble human BilIN and ICPN in the EHBD and GB, respectively. Mechanistically, c-Myc contributes to tumorigenesis, whereas the TGF-β pathway inhibits it.

In summary, concurrent activation of the Kras and Wnt pathways in the extrahepatic biliary system induced ICPN and BilIN, which can progress to biliary cancer (Figure 1). This study provides the first novel GEM that recapitulates human ICPN and BilIN, establishing them precancerous lesions. This work shows how dysregulation of canonical cell growth pathways drives precursors to biliary cancers and identifies several molecular vulnerabilities as potential therapeutic targets in these precursors to prevent oncogenic progression. Mechanistically, c-Myc contributed to tumorigenesis, whereas the TGF-β pathway inhibited it. Consistent with the mouse data, the KRAS, WNT signaling, c-MYC, and TGF-β pathway were activated in human ICPNs.

Recently, it was reported that CCA has a complex mutational landscape [11, 16]. However, little is known about the molecular biological significance of each genetic alternation. To develop novel preventive and therapeutic approaches for extrahepatic biliary cancer, it is also important to clarify the role of other altered genes by using a GEM model and/or human samples.
